# Motor Imagery Brain–Computer Interface (MI-BCI)-Assisted Upper Limb Neurorehabilitation for Acute Stroke During Inpatient Rehabilitation: A Prospective Feasibility Study with Economic Evaluation Protocol

**DOI:** 10.3390/jcm15145692

**Published:** 2026-07-20

**Authors:** Ravi Shankar, Yu Tung Lo, Chin Lay Fong, Nicole Keong, Karen Sui Geok Chua

**Affiliations:** 1 Clinical Research & Innovation Office, Tan Tock Seng Hospital, National Healthcare Group (NHG) Health, Singapore 138543, Singapore; 2 Department of Neurosurgery, National Neuroscience Institute, Singapore 308433, Singapore; jacklo@nus.edu.sg (Y.T.L.); nicole.keong.c.h@singhealth.com.sg (N.K.); 3 Duke-NUS Medical School, Singapore 169857, Singapore; 4 Rehabilitation Research Institute of Singapore, Nanyang Technological University, Singapore 639798, Singapore; karen.chua@nhghealth.com.sg; 5 National Healthcare Group (NHG) Health, Institute of Rehabilitation Excellence, Tan Tock Seng Hospital Rehabilitation Centre, Singapore 138543, Singapore; lay.fong.chin@nhghealth.com.sg; 6 Yong Loo Lin School of Medicine, National University of Singapore, Singapore 119077, Singapore; 7 Lee Kong Chian School of Medicine, Nanyang Technological University, Singapore 639798, Singapore

**Keywords:** brain–computer interface, stroke rehabilitation, neuroplasticity, motor imagery, functional electrical stimulation, upper limb, feasibility study, cost-effectiveness

## Abstract

**Background:** Stroke is a leading cause of neurological disability worldwide, with upper limb impairment affecting approximately 70% of survivors and only 5–20% achieving complete dexterity recovery at six months. Brain–computer interface (BCI) neurorehabilitation decodes motor intentions from electroencephalographic (EEG) signals to deliver synchronized functional electrical stimulation (FES) and virtual reality feedback, creating a closed-loop neurofeedback system that reinforces motor learning. While existing evidence supports BCI efficacy and safety in chronic stroke, its feasibility, safety, and cost-effectiveness during the acute and subacute phase (2 to 12 weeks post-stroke), when neuroplasticity is heightened, remain underexplored. Furthermore, there is a paucity of data regarding preliminary health economic analyses for BCI rehabilitation in acute stroke rehabilitation settings. **Methods:** This prospective, open-label, single-arm pragmatic feasibility pilot trial will recruit 12 patients with hemorrhagic or ischemic stroke (2–12 weeks post-stroke) undergoing inpatient rehabilitation from a public healthcare institution. Up to 15 sessions of BCI-rehabilitation of 30 min each using the recoveriX system will be supervised by a trained therapist or clinical research assistant (4–5 sessions/week over 3–4 weeks), followed by standard occupational therapy within 30–60 min of BCI-rehabilitation. Primary outcomes assessing feasibility and adherence include eligibility and recruitment rate (%/screened); tolerability using self-rated System Usability Scale (SUS) score; within-session adherence > 80%/240 trials, summated for completed trials per patient; programme completion number > 80% of scheduled (>12/15) sessions; and training-related adverse events per patient ≤ 17% (≤2/12 sessions). Secondary outcome measures include clinical efficacy by arm impairment scale using hemiplegic Upper Limb Fugl–Meyer Motor Assessment (FMA-UE), hand function using Action Research Arm Test (ARAT), admission and discharge functional status (Functional Independence Measure-FIM (18–126), Modified Barthel Index-MBI (0–100), stroke impact scale (SIS_3.0), arm, participation domains), and economic analysis. All outcomes will be measured by trained therapists/researchers at baseline week 0, week 3–4 (post-BCI-rehabilitation), and week 12 and 24 (follow-up). BCI-rehabilitation EEG-derived electrophysiological correlates of recovery will be extracted to better understand participant progress over time. An incremental cost-utility analysis will compare the BCI-rehabilitation participants against propensity-matched historical controls from the TTSH stroke rehabilitation registry (2017 to 2025), stratified by baseline motor severity. **Discussion:** This study will provide preliminary evidence on feasibility, tolerability, safety, clinical efficacy, and cost-effectiveness of early BCI-rehabilitation in acute/subacute stroke to better inform clinicians on its implementation.

## 1. Background

Stroke represents a major global health burden, ranking as the second leading cause of mortality and the third leading cause of combined mortality and disability as measured by disability-adjusted life years (DALYs) worldwide [1,2]. In Singapore, the incidence of stroke increased substantially over the last decade. According to data from the Singapore Stroke Registry, there were 9702 stroke episodes in 2022, representing a more than 50% increase compared with 6367 episodes reported in 2012 [3]. This rise reflects a growing stroke burden in the nation [4]. Among the multiple consequences of stroke, upper limb impairments are particularly prevalent and debilitating, with approximately 70% of stroke survivors demonstrating altered arm function, 40% maintaining persistent functional disability, and only 5–20% achieving complete dexterity recovery at 6 months post-stroke [5,6].

Current rehabilitation options for stroke-related UL impairment include conventional occupational therapy (OT), constraint-induced movement therapy (CIMT), robot-assisted therapy (RAT), and non-invasive brain stimulation techniques such as transcranial magnetic stimulation (TMS) and transcranial direct current stimulation (tDCS) [7,8,9]. Conventional OT remains the cornerstone of standard post-stroke arm and hand rehabilitation, focusing on task-specific training and activities of daily living retraining; however, its benefits are contingent on the patient’s capacity for active voluntary movement, limiting its utility in patients with severe motor deficits. CIMT, which involves intensive use of the paretic limb with restraint of the unaffected limb, has demonstrated meaningful and enduring efficacy in patients with at least some residual distal motor function but is poorly tolerated in the acute setting, requires strict inclusion criteria and patient selection, and is unsuitable for patients with complete plegia [7]. RAT offers the advantage of delivering high-repetition, task-specific training with reduced therapist burden possible through minimally supervised care models, but widespread access is often limited by prohibitive cost and complexity of devices, inconsistent adoption related to cost-effectiveness data, and organizational factors impacting intensive service delivery mimicking research protocols [8]. Non-invasive brain stimulation serves as a promising adjunct by modulating cortical excitability; however, functional recovery is modest and heterogeneous across trials, with uncertain durability [9]. All conventional approaches lack a direct, real-time link between a patient’s motor intention and the resulting limb movement, limiting Hebbian-based neuroplastic changes, particularly in patients with severe corticospinal tract disruption [10].

Brain–computer interface (BCI) addresses this lack of a closed-loop system connecting volitional intent with limb movement [11,12]. BCI records neural signals, decodes motor intents, and uses decoded signals to control either external devices (end effector robots or neuroprostheses or virtual reality (VR platforms), or internal effectors (functional electrical stimulation of the patient’s muscles [13,14], thus reestablishing volitional control of their paretic limbs through motor imagery via electroencephalographic (EEG) signal decoding or MI-EEG feedback [15] and restoring temporal dependence between cortical (presynaptic) and end effector (postsynaptic) neurons. The resulting successful MI-EEG triggering the end-effector limb movement in turn generates ascending sensory perception, completing a closed-loop biofeedback system that reinforces motor learning [16,17].

Recent systematic reviews and meta-analyses have demonstrated that BCI-based rehabilitation produces clinically and statistically significant improvements in upper extremity motor function [18,19,20]. Locally, Ang et al. (2011) conducted a large clinical study demonstrating that stroke patients could successfully use EEG-based motor imagery BCI, establishing feasibility in this population [21]. Subsequent research by Várkuti et al. (2013) showed that resting state changes in functional connectivity correlated with movement recovery following BCI and robot-assisted upper-extremity training [22]. Ang et al. (2015) in two randomized controlled trials comparing duration-matched controls with either RAT or BCI-RAT paired with either shoulder–hand or wrist–hand end-effector robots, demonstrated the efficacy of MI-EEG-RAT in chronic stroke patients with significant pre/post-training effects without significant inter-group differences between conventional UE rehabilitation controls, robotic only, and BCI-RAT intervention groups [21,23]. Furthermore, BCI-RAT groups were associated with high levels of safety, and within group impairment reductions were sustained till week 24 follow-up, thus providing an important foundation for extending BCI research into the subacute/chronic stroke inpatient populations [21,23].

**Figure 1:**
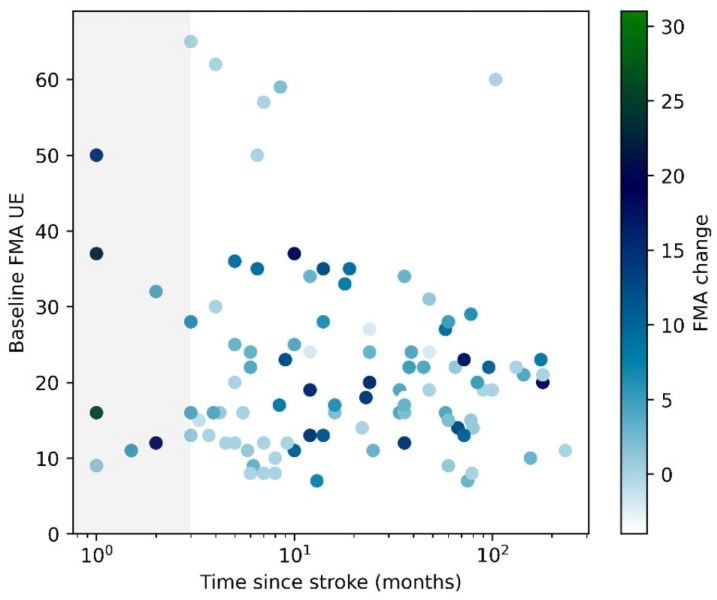
The distribution of time since stroke at the initiation of BCI-based rehabilitation against baseline upper limb impairment, drawn from our published individual patient data meta-analysis of existing BCI stroke studies [24]. Each point represents one patient; the horizontal axis shows time since stroke in months on a logarithmic scale, and the point color denotes the change in Fugl–Meyer Assessment Upper Extremity score following BCI-based therapy. The shaded band marks the first three months after stroke. The overwhelming majority of patients in existing studies were treated in the chronic phase, with only 8.8% commencing BCI-based rehabilitation within the first three months. This illustrates the paucity of evidence for the acute and early subacute window that the present study seeks to address.

Existing studies, however, involved predominantly chronic stroke patients. In our recent meta-analysis of predominantly chronic stroke cohorts, BCI rehabilitation produced a mean improvement of 5.23 points on the Fugl–Meyer Assessment Upper Extremity (95% confidence interval 3.85 to 6.61; total score range 0 to 66, with higher scores indicating less impairment), exceeding the established minimal clinically important difference of 4 to 6 points for the FMA-UE [24]. We hypothesize that there may potentially be greater benefits when BCI is applied during the acute/subacute phase when neuroplasticity is heightened. Notably, an overview of systematic reviews by Liu et al. (2025) concluded that BCI-combined treatment improves upper limb motor function and quality of daily life, with effects particularly pronounced in the subacute phase [25]. In acute and subacute stroke, several studies have alluded to moderate quality evidence and high safety profile and efficacy of BCI when coupled with end effectors such as FES, VR, or robots, achieving mean FMA-UE difference of 3.69–5.02 albeit with less robust gains for ADL independence. Subgroup analyses indicated that interventions < 30 days of stroke for <3 weeks may yield greater benefits, in particular paired with FES. Optimal training parameters suggested daily sessions of 20–90 min or 2–5 times weekly for 3–4 weeks [26,27] (Figure 1).

However, practical implementation of BCI technology within acute inpatient hospital settings presents logistical challenges and must also be carefully evaluated. The setup of EEG headsets with wet electrodes requires trained personnel and can add at least 10–15 min of preparation time per session, including set-down and scalp cleansing after MI-BCI to achieve adequate electrode impedance [28,29].

Lastly, economic data remain scarce, creating barriers to widespread implementation in healthcare systems [30]. Understanding the incremental cost-effectiveness of BCI rehabilitation compared to standard care is essential for informing policy decisions regarding technology adoption and reimbursement. This study therefore incorporates a prospective economic evaluation alongside the clinical feasibility assessment to generate preliminary cost-effectiveness evidence.

This protocol addresses three gaps that, to our knowledge, have not previously been examined together in the BCI stroke rehabilitation literature. First, almost all existing BCI evidence is derived from chronic stroke survivors who have already plateaued after conventional rehabilitation, whereas the acute and early subacute inpatient phase, when neuroplasticity is theoretically greatest, remains largely untested. This phase also presents distinct patient selection challenges because reduced attention, fatigability, and medical instability are common early after stroke and may constrain who can engage with an attention-dependent motor imagery paradigm; this consideration directly informs our cognitive and tolerance-based eligibility criteria. Second, no existing study has evaluated the translation of BCI into routine ward practice through an implementation science lens despite well-recognized barriers relating to electrode set-up time, staffing, and device complexity. Third, no prior BCI rehabilitation study has reported a preliminary health economic evaluation, leaving decision-makers without cost-effectiveness evidence to guide adoption. In addition, the acute and severe stroke population has a particular need for, yet limited published safety data exist on, closed-loop BCI; systematic capture of adverse events such as fatigue, skin reactions, and seizure risk in this more vulnerable group is therefore a deliberate focus of this study.

The aims of this study protocol are to (1) establish the feasibility, usability, and safety of implementing BCI-based rehabilitation during the inpatient acute/early subacute stroke phase; (2) evaluate clinical efficacy outcomes with regards to motor impairment scales and quality of life measures; (3) characterize the electrophysiological correlates of motor recovery during this critical neuroplasticity window; and (4) conduct preliminary health economic evaluation.

## 2. Methods

### 2.1. Study Design

This is a prospective, open-label, single-arm pragmatic feasibility pilot trial employing consecutive sampling with independent and periodic outcome assessments, incorporating a preliminary cost analysis using historical controls. A CONSORT-compliant participant flow diagram will be included in the results publication. The study has been designed following the Standard Protocol Items: Recommendations for Interventional Trials (SPIRIT) guidelines and will be reported according to Consolidated Standards of Reporting Trials (CONSORT) extension for pilot and feasibility trials [31,32]. The economic evaluation will follow the Consolidated Health Economic Evaluation Reporting Standard (CHEERS) guidelines [33].

### 2.2. Study Setting

Participants will be recruited from the inpatient rehabilitation unit of Tan Tock Seng Hospital (TTSH)-Integrated Care Hub (TTSH-ICH), which receives referrals from the acute stroke units of National Neuroscience Institute (NNI), Singapore.

### 2.3. Eligibility Criteria

**Inclusion criteria are as follows:** adults aged 21–80 years with confirmed hemorrhagic or ischemic stroke on neuroimaging, stroke onset within 2–12 weeks; demonstrated voluntary finger flexion or extension by Oxford Medical Research Council (MRC) grade 0–4/5, Montreal Cognitive Assessment (MoCA) score > 20/30; ability to understand written and spoken instructions; and stable medical and neurological status as determined by the clinical team [34]. Because the closed-loop paradigm depends on integrating afferent (proprioceptive and visual) feedback with efferent motor intent, lesion location will be documented from neuroimaging; participants with predominantly cerebellar or brainstem lesions—who may retain intact primary sensation yet have impaired updating of internal sensorimotor (forward) models, that is, impaired predictive coding—will remain eligible but will be flagged a priori. For such participants the intervention explicitly leverages the multimodal, error-corrective feedback intrinsic to the recoveriX loop (synchronized FES-evoked movement together with visual avatar feedback) as an externally supplied source of sensory prediction; their decoding performance and clinical response will be examined as an exploratory subgroup to determine whether impaired predictive coding attenuates BCI benefit and should inform eligibility for a future definitive trial. Because the motor-imagery-related event-related desynchronization (ERD) used for BCI classification is recorded over central sensorimotor electrodes (FC3–CP4) rather than posterior/occipital sites, cerebellar lesions are not expected to directly impair this signal; ERD magnitude and BCI performance in this subgroup will nonetheless be examined exploratively as above.

**Exclusion criteria are as follows:** previous diagnosis of stroke; implanted pacemaker or continuous infusion pump; history of seizures or epilepsy; previous neurosurgical procedures, medical instability as determined by the study team; severe blindness; anticipated life expectancy less than 6 months; hemodialysis requirement; inability to follow one-step commands; severe aphasia or dysarthria; severe arm/hand pain (Visual Analog Scale > 4/10); severe hemianesthesia or spasticity in the paretic arm (Modified Ashworth Scale greater than 2, out of 4); fixed shoulder or finger joint contractures; active arthritis in any upper limb joint; inability to tolerate upright supported sitting for at least 45 min; and disorders of consciousness or altered arousal states precluding active participation.

Written informed consent will be obtained from all participants or their legally appointed representatives, the latter group applicable to those with MoCA 21–25/30.

### 2.4. Sample Size

Based on comparable feasibility studies in BCI stroke rehabilitation literature and recommendations from our recent meta-analysis [24], a sample of 10 subjects will be sufficient for this feasibility study. Accounting for a 20% dropout rate (*n* = 2), the final proposed sample size is 12 participants. Recruitment will aim to stratify the sample by stroke type in an approximate ratio of 40% hemorrhagic (ICH) to 60% ischemic (infarct) stroke, consistent with local case-mix.

This sample size follows established guidance for pilot and feasibility trials, in which the objective is to estimate feasibility parameters and their variability rather than to test efficacy; widely cited recommendations propose approximately 12 participants for pilot studies [35,36], and a sample of 10–12 is adequate to estimate a feasibility proportion of about 80% (for example recruitment or adherence) with a 95% confidence interval half-width of roughly ±20%. A formal efficacy-based power calculation is therefore not appropriate for the present feasibility endpoints. Nonetheless, to inform the design of a future definitive trial, an indicative a priori power calculation was performed in G*Power (version 3.1). Anchoring conservatively on 6 points—the lower end of the FMA-UE minimal clinically important difference range established largely in chronic stroke (4–6 points)—acute-stroke-specific estimates are reportedly higher, approximately 7–10 points, but 6 points was retained as a conservative estimate and the pooled effect implied by our meta-analysis (mean change 5.23 points, standard error 0.70; approximate standard deviation 3.4 in the analyzed cohorts) [24]; a two-sided paired within-group comparison (α = 0.05, power = 0.80) would require approximately 6–8 participants to detect a change of the minimal clinically important difference magnitude, whereas a two-arm parallel comparison designed to detect a 4-point between-group difference at an assumed standard deviation of 6 (Cohen’s d ≈ 0.67) would require approximately 37–46 participants per arm. The variance and recruitment-rate estimates generated by the present feasibility study will be used to refine these calculations and to power the subsequent randomized controlled trial.

### 2.5. Study Hypotheses

This feasibility pilot trial will test the following primary hypotheses:

1. Usability: Mean System Usability Scale (SUS) score > 70 in 75% [9] of participants.

2. Session adherence: 75% [9] of participants completing > 24 min (>80%) of each scheduled 30 min BCI session.

3. Program completion: >75% [9] of enrolled participants completing 80% planned sessions (12/15). Commencement of occupational therapy sessions within 30–60 min of completion of MI-EEG. Inpatient drop out < 20% (<3) excluding unanticipated medical/neurosurgical reasons or unplanned early discharge. Participants who are unexpectedly discharged early or become medically unwell will be recorded as protocol deviations and reasons documented.

4. Safety: Training-related adverse events per total delivered sessions ≤ 17% (≤2/12 sessions). These are described as headache or pain from cap or FES application by visual analogue scale (VAS > 5/10, skin changes (from cap or FES electrodes, blurring of vision, from computer eye fatigue, seizures, mental fatigue).

Secondary hypotheses include the following:

(i) Clinical efficacy and post-stroke dependency measured by FMA-UE, ARAT, FIM, MBI.

(ii) Health-related quality of life by SIS-3.0 (arm, hand, activities of daily living-ADL, societal participation, global rating of recovery, domains 1, 5, 7, 8, 9).

(iii) Preliminary cost-effectiveness estimates stratified by baseline severity, including program cost comparisons and the incremental cost-effectiveness ratio (ICER).

### 2.6. Intervention

BCI-based rehabilitation will be delivered using the recoveriX system (g.tec medical engineering GmbH, Austria), a commercially available FDA-approved BCI platform that has been validated in stroke and multiple sclerosis [28,37]. The recoveriX system integrates three key components: (1) a wet-electrode EEG head cap for neural signal acquisition, (2) signal processing software that automatically calibrates and decodes motor imagery patterns, and (3) a functional electrical stimulator (FES) that delivers peripheral muscle stimulation synchronized with detected motor intent [38].

**Figure 2:**
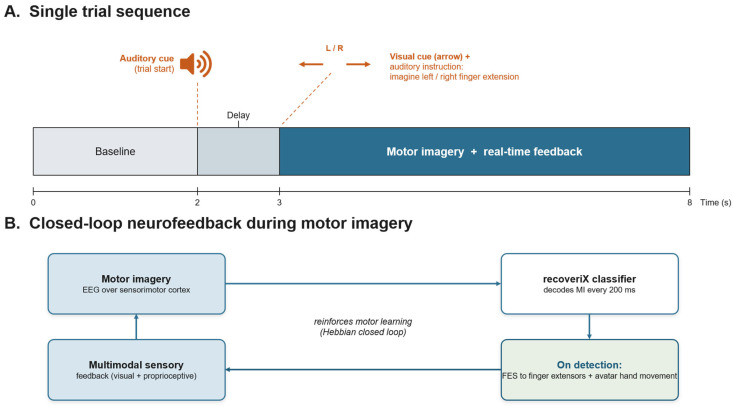
A schematic of a single recoveriX motor imagery brain–computer interface trial. Each trial comprises a 2 s baseline period, an auditory cue signaling trial initiation, a 1 s delay, and a visual cue (an animated arrow) presented with a concurrent auditory instruction indicating whether to imagine left or right finger extension, followed by 5 s of motor imagery with real-time feedback. Motor imagery is decoded every 200 ms; upon detection, functional electrical stimulation is delivered to the forearm finger extensor muscles while an on-screen avatar displays the corresponding hand movement, providing synchronized multimodal sensory feedback.

**Training Protocol:** Participants will undergo up to 15 BCI rehabilitation sessions over 3–4 weeks (4–5 × 30 min sessions per week), supervised by trained therapists or clinical research assistants. Each session comprises three blocks of 80 trials (240 trials total). Individual trials follow a structured sequence (Figure 2): 2 s baseline period, auditory cue signaling trial initiation, 1 s delay, visual cue (animated arrow) with auditory instruction indicating whether to imagine left or right finger extension, followed by 5 s of motor imagery with real-time feedback. The motor imagery employed is kinesthetic, first-person imagery of a discrete, non-goal-directed movement—specifically, imagining the sensation of extending the fingers and opening the hand on the cued side—rather than goal-directed, object-oriented actions such as reaching to grasp an object. This reflects the validated two-class recoveriX paradigm and the need for a temporally precise, repeatable motor template that can be reliably decoded and synchronized with functional electrical stimulation; goal-directed, object-oriented practice is instead delivered during the subsequent conventional occupational therapy block. Motor imagery detection is assessed every 200 milliseconds; upon detection, the FES stimulates the forearm finger extensor muscles while an avatar on screen displays corresponding hand movement, providing multimodal sensory feedback [39].

**FES Parameters:** Electrode skin pads will be placed over the forearm finger extensor muscle groups. Electrical stimulation will be delivered at a pulse frequency of 50 Hz with a pulse width of 300 microseconds. Stimulation intensity (in milliamperes) will be individually titrated to produce visible wrist and/or finger extension without pain or muscle spasms.

**Concomitant Therapy:** Following each recoveriX session (approximately 30 min), participants will receive 60 min of standard-of-care conventional occupational therapy within 30–60 min of BCI training completion, resulting in a total therapy time of 90 min per session. This represents additional therapy (approximately 30 min) compared to standard care [40]. We acknowledge that as designed, this adjunctive schedule confounds the specific effect of closed-loop BCI neurofeedback with the effect of additional therapy time so that any functional gain cannot be attributed unambiguously to the brain–computer interface rather than to a greater rehabilitation dose. An adjunctive design was nonetheless chosen for pragmatic and ethical reasons in this acute inpatient population, in which withholding standard therapy in order to time-equate the arms would be inappropriate and the primary aim is feasibility rather than efficacy. To mitigate this confounder, we will (i) prospectively record the total upper limb therapy dose (in minutes and repetitions) for both BCI participants and historical controls, (ii) enter total therapy time as a covariate in exploratory effectiveness and cost analyses, and (iii) report the incremental cost of the additional therapist time separately from device-related costs. For the future definitive trial, a dose-matched (time-equated) active comparator—delivering an equivalent duration of conventional or sham/attention-controlled therapy—is planned so that the independent contribution of the closed-loop BCI can be isolated. Standard inpatient multidisciplinary stroke rehabilitation therapies will continue throughout the inpatient rehabilitation period. To prevent potential contamination of therapeutic effects, standard non-invasive upper limb FES over the forearm finger extensor muscle groups will not be applied during the inpatient stay where possible.

## 3. Outcome Measures

Outcome assessments will be conducted at four time points: baseline (week 0), end of intervention period of 15 sessions (week 3–4), and follow-up assessments at weeks 12 and 24 (week 24 corresponds to approximately six months post-baseline). To evaluate the longer-term retention of motor recovery and the durability of any BCI-induced electrophysiological changes, an additional follow-up will be conducted at week 52 (12 months), comprising the clinical (FMA-UE, ARAT, FIM, MBI) and quality-of-life (EQ-5D-5L, SIS-3.0) measures and, where the participant is able to attend, a repeat EEG/BCI recording; remote or telephone administration of the patient-reported outcomes will be offered to minimize attrition at this extended time point. All clinical assessments will be performed by trained independent assessors (occupational therapists or research assistants) (Table 1).

**Feasibility Outcomes:** As this is a pragmatic feasibility pilot trial, we will systematically test pre-specified feasibility benchmarks: (1) System Usability Scale (SUS) score >70 in ≥75% [9] of participants, assessed weekly; (2) within-session adherence ≥ 80% (completing ≥24 min per 30 min session) in ≥75% [9] of participants; (3) program completion in ≥75% [9] of participants, each completing ≥ 80% (≥12 of 15) of scheduled sessions; and (4) recorded adverse events related to training ≤ 17% (≤2 of 12 sessions) [41]. Secondary feasibility metrics include clinical efficacy and cost-utility analysis.

**BCI Performance Metrics:** Variables relating to recoveriX BCI system performance will be collected including motor imagery classification accuracy, BCI compatibility index, response times, and session-by-session variability in decoding performance. These metrics will provide insight into the reliability of the BCI system in this patient population and potential predictors of clinical response. These technical metrics constitute a primary feasibility domain and are defined a priori as follows. Motor imagery classification accuracy is the proportion of cued trials in which the online classifier correctly identifies the intended (left or right) hand for the two-class paradigm, computed per session; a session is considered to demonstrate adequate BCI control when accuracy exceeds the participant-specific permutation-derived chance level and meets the conventional binary BCI control benchmark of at least 70%. The true positive rate is the proportion of cued trials in which motor imagery is detected and triggers functional electrical stimulation on the correct side. The BCI compatibility index is the recoveriX system index of how reliably a participant’s motor imagery can be decoded. Session-to-session reliability is the within-participant variability in classification accuracy across the training course. At the cohort level, we will report the proportion of participants achieving the 70% accuracy benchmark (a measure of BCI literacy in acute and subacute stroke) and the trajectory of accuracy over successive sessions.

### 3.1. Signal Acquisition and Processing

EEG signals will be recorded throughout each therapy session using the recoveriX system’s integrated amplifier (g.USBamp) via 11 active wet electrodes positioned over sensorimotor cortex (10–20 system: FC3, FCz, FC4, C3, C1, Cz, C2, C4, CP3, CPz, CP4, and additional reference channels), sampled at 256 Hz with online 0.5–30 Hz bandpass and 50 Hz notch filtering. In addition, and in response to reviewer feedback, two occipital derivations (O1 and O2) will be recorded where the amplifier channel configuration permits so that the dominant posterior (alpha) rhythm can be monitored continuously as an on-line index of arousal. This is important because early post-stroke patients frequently exhibit fluctuating vigilance (drowsiness or stupor), which can confound an attention-dependent motor imagery paradigm; trials or sessions recorded during posterior-rhythm-confirmed drowsiness will be flagged and, where appropriate, excluded from efficacy analyses (see quality-control pipeline below). Given that the inclusion criterion of MoCA > 20/30 and exclusion of participants with disorders of consciousness or altered arousal states already limit enrollment of patients prone to clinically significant drowsiness, this concern is expected to be largely mitigated at the eligibility stage; posterior-rhythm monitoring is retained as an additional session-level safeguard. Post hoc signal processing will be performed using Python with established neuroscientific analysis packages. Processing pipeline will include frequency filtering (bandpass filters isolating delta, theta, alpha/mu, beta, and gamma bands), spatial filtering using Common Spatial Pattern algorithms, and feature extraction for machine learning analysis [42]. We will employ Linear Discriminant Analysis (LDA) and/or other classification approaches to identify electrophysiological features correlated with motor recovery trajectories. CSP+LDA was selected, as it is the validated reference pipeline within recoveriX and remains robust at the small per-session sample sizes typical of clinical BCI use. Successful motor imagery generation will be verified per session via the recoveriX BCI compatibility index and cross-validated classification accuracy against permutation-derived chance level, with FES triggered only when the classifier detects motor imagery for the cued side. To address EEG contamination from concurrent FES, stimulation epochs will be excluded from online classifier updates, and offline analyses will apply standard stimulation-artifact removal (notch filtering at the stimulation frequency and its harmonics, with ICA-based rejection of stimulation-locked components).

To standardize electrophysiological data quality and support reproducibility, a pre-specified signal quality-control and preprocessing pipeline will be applied. Prior to every session, electrode–skin impedance will be verified and maintained below 10 kΩ (target < 5 kΩ) for all active electrodes, with re-gelling or re-seating of electrodes and re-testing until this threshold is achieved; impedance values will be logged for each session. Continuous EEG will be segmented into cue-locked epochs, and a uniform artefact-handling pipeline will comprise (i) band-pass (0.5–30 Hz) and 50 Hz notch filtering; (ii) automated rejection of epochs exceeding ±100 µV or containing flat or saturated (railing) segments; (iii) independent component analysis (extended Infomax) with removal of components classified as ocular (blink or saccade), electromyographic, electrocardiographic, or functional-electrical-stimulation-locked artefact, using a combination of an automated classifier (for example ICLabel) and blinded expert visual review; and (iv) exclusion of any session in which fewer than 60% of cued trials survive artefact rejection or in which the posterior alpha rhythm indicates sustained drowsiness. All data exclusions and their reasons will be pre-specified and reported transparently to support reproducibility of the electrophysiological biomarker analyses.

A priori electrophysiological biomarkers of motor recovery, and their association with clinical change, will be defined and tested in a pre-specified manner. The primary candidate biomarkers are (i) the magnitude of event-related desynchronization and synchronization (ERD/ERS) in the contralesional and ipsilesional mu (8–13 Hz) and beta (13–30 Hz) bands during motor imagery; (ii) the hemispheric laterality index of sensorimotor ERD; (iii) the CSP-LDA classification accuracy and its session-to-session trajectory; and (iv) the recoveriX BCI compatibility index. The principal clinical anchor will be the change in FMA-UE from baseline to week 3–4, with change in ARAT as a secondary anchor. For each biomarker, both the baseline value and the within-training slope will be related to clinical change using Spearman rank correlation coefficients and, where the sample permits, robust linear regression adjusting for baseline motor severity, with 95% confidence intervals reported throughout. Given the exploratory sample size, these biomarker–outcome associations are explicitly hypothesis-generating; they will be interpreted descriptively, with Benjamini–Hochberg control of the false discovery rate, rather than as confirmatory tests, and will be used to nominate candidate predictive biomarkers for a future definitive trial.

As an exploratory predictor of treatment response and following reviewer suggestion, sensorimotor ERD will additionally be recorded during a brief action-observation task at baseline, in which the participant watches a filmed version of the to-be-performed hand movement. Motor imagery and action observation engage a largely shared frontoparietal network—the putative human mirror mechanism [43]—and a robust ERD elicited while observing a to-be-performed action has been associated with a favorable response to motor rehabilitation after stroke [44,45]. Baseline action-observation ERD will therefore be examined as a candidate stratifying biomarker and, potentially, as an eligibility-refining criterion for a future definitive trial rather than as a mandatory enrolment criterion in the present feasibility study.

### 3.2. Economic Evaluation

An incremental cost–utility analysis (CUA) will be conducted alongside the feasibility study to generate exploratory cost data of BCI rehabilitation compared to standard care, acknowledging the limited precision afforded by the single-arm design. The economic evaluation will adopt a healthcare system perspective over a 24-week time horizon, consistent with the clinical follow-up period.

To keep the economic evaluation focused, it is organized around two scopes: (1) the within-trial 24-week cost–utility analysis from a healthcare-system perspective described above and (2) a secondary long-term extrapolation. For the latter, a cohort state-transition (Markov) model, structured around post-stroke functional health states (for example, FMA-UE severity together with functional dependency) and an absorbing death state, will project costs and quality-adjusted life years over 2-year and 5-year horizons, using an annual cycle with half-cycle correction and discounting at 3% per annum (0% and 5% in sensitivity analysis), consistent with Singapore health-technology-assessment norms. Transition probabilities and utilities will be derived from the trial, the TTSH registry, and the published literature; a societal perspective incorporating productivity losses and informal caregiver time will be explored as a sensitivity analysis rather than as a primary scope. As it remains uncertain whether clinical gains from BCI-rehabilitation are sustained into the chronic phase beyond the trial follow-up period, all estimates extrapolated beyond 24 weeks will be treated as exploratory and interpreted with caution. Structural and parameter uncertainty will be characterized through deterministic and probabilistic sensitivity analyses, reported in accordance with CHEERS 2022.

### 3.3. Comparator Group: Historical Controls

Given the single-arm feasibility design, the comparator group will be derived from the TTSH stroke rehabilitation registry (2017–2025). Historical controls will be propensity-matched to BCI participants based on (1) baseline FMA-UE score, (2) age, (3) stroke type (hemorrhagic vs. ischemic), and (4) time post-stroke at rehabilitation admission (2–12 weeks). To reduce residual confounding, the propensity model will additionally include sex, baseline functional status (admission FIM and MBI), comorbidity burden (Charlson Comorbidity Index), clinically relevant concomitant medications (for example antispasticity and mood-active agents), the total inpatient rehabilitation therapy dose, and the calendar year (era) of admission. Historical control data will be obtained from an institutional inpatient stroke rehabilitation cohort assembled between 2022 and 2025 (more than 100 patients), for which baseline and discharge EQ-5D-5L utilities and FMA-UE scores are available to support propensity matching and quality-adjusted life year estimation. Corresponding inpatient rehabilitation cost data will be obtained from hospital administrative and costing databases. FMA-UE categories may use severe impairment (FMA-UE 0–19) and moderate-to-mild impairment (FMA-UE 20–47). This approach has been previously validated in the TTSH setting for evaluating upper extremity recovery outcomes [40]. Matching will be performed using nearest-neighbor propensity score matching with a caliper of 0.2 standard deviations to ensure adequate balance on baseline covariates. The matching ratio will be 1:3 (BCI/control) to maximize statistical power while maintaining adequate matching quality. Balance diagnostics will be assessed using standardized mean differences (target < 0.1 for all covariates) and variance ratios. To limit temporal confounding arising from the eight-year registry window (2017–2025)—over which ward staffing, rehabilitation protocols, and case-mix may have changed—the primary comparator analysis will be restricted to the contemporaneous 2022–2025 inpatient cohort, calendar year will be included as a matching and adjustment covariate, and models will additionally be fitted with admission era as a fixed effect; the influence of the matching window will be examined in sensitivity analyses that progressively restrict controls to narrower, more recent time bands. Sensitivity analyses will explore alternative matching specifications, including different caliper widths and matching algorithms (genetic matching, coarsened exact matching) to assess robustness of findings to matching choices. If precise stroke onset dates are unavailable for historical controls, hospital arrival date will be used as a proxy, consistent with Singapore Stroke Registry methodology.

### 3.4. Cost Data Collection

Costs will be estimated from the healthcare system perspective and will include both intervention costs and downstream healthcare resource utilization. All costs will be reported in 2025 Singapore dollars (SGD) (Table 2).

### 3.5. Cost Perspective and Therapy Time Differential

The BCI intervention is modeled as additional adjunctive therapy in addition to standard of care rather than a substitute for standard care. Under this assumption (Scenario A), BCI participants receive 30 min of BCI training plus 60 min of standard occupational therapy per session (90 min total), whereas standard care patients receive approximately 60 min of occupational therapy per session based on TTSH rehabilitation protocols [40]. The incremental cost of BCI therefore includes both equipment-related costs and the additional 30 min of therapist time per session. Where available, actual billed charges from hospital administrative databases will be used to estimate healthcare costs. For intervention-specific costs not captured in routine billing (e.g., BCI equipment depreciation, additional therapist time), unit costs will be derived from institutional costing data. This pragmatic approach balances data availability with accuracy of cost estimation. A sensitivity scenario modeling partial substitution—approximately 25%, rather than 50%, of within-session supervision withdrawn from the midpoint of the training course following an initial supervised orientation period, noting this proportion may vary in practice—will be examined to explore conditions under which a favorable ICER may be achievable.

### 3.6. Effectiveness Measure

The primary effectiveness measure for the cost-utility analysis will be quality-adjusted life years (QALYs), calculated from EQ-5D-5L utility scores collected at baseline, week 3–4, week 12, and week 24. Singapore-specific value sets will be applied to convert EQ-5D-5L health states to utility weights [46]. QALYs will be calculated using the area-under-the-curve method with linear interpolation between assessment points. For historical controls, EQ-5D-5L data will be extracted from the TTSH registry where available; if unavailable, utility values will be mapped from functional outcomes (FMA-UE, Barthel Index) using validated mapping algorithms [47].

### 3.7. Cost-Effectiveness Analysis

The incremental cost-effectiveness ratio (ICER) will be calculated as the difference in mean costs between BCI and standard care groups divided by the difference in mean QALYs: ICER = (Cost_BCI—Cost_Control)/(QALY_BCI—QALY_Control). Results will be interpreted against the Singapore willingness-to-pay threshold of approximately SGD 50,000–100,000 per QALY gained. A secondary cost-effectiveness analysis will calculate the cost per point improvement in FMA-UE to provide clinically interpretable metrics alongside the cost-utility analysis.

### 3.8. Stratified Analysis by Baseline Severity

Given evidence that baseline motor severity substantially influences recovery trajectories [40,48], the economic analysis will be stratified by baseline FMA-UE severity using validated cutoffs: (1) severe impairment (FMA-UE 0–19) and (2) moderate-to-mild impairment (FMA-UE 20–47) [48]. These strata reflect distinct recovery trajectories and will identify subgroups for whom BCI rehabilitation may be most cost-effective. Given the small sample size (*n* = 12), these subgroup analyses are strictly exploratory, and results should be interpreted with caution.

### 3.9. Sensitivity and Uncertainty Analysis

Deterministic sensitivity analyses will be conducted to assess the robustness of results to parameter uncertainty, including variations in (1) equipment costs (purchase vs. rental models), (2) therapist time costs, (3) discount rates (0% and 5% annually), and (4) utility mapping assumptions for historical controls. Probabilistic sensitivity analysis using Monte Carlo simulation (5000 iterations) will characterize joint parameter uncertainty and generate cost-effectiveness acceptability curves showing the probability of BCI being cost-effective across a range of willingness-to-pay thresholds. An additional scenario analysis will model partial supervision—approximately 25%, rather than 50%, of sessions unsupervised from the midpoint of training following initial orientation, acknowledging this proportion may vary in practice—to assess the minimum substitution level required for a favorable incremental cost-effectiveness ratio.

### 3.10. Limitations of Economic Evaluation

Several limitations of the exploratory cost analysis should be acknowledged. First, the use of historical controls rather than a concurrent randomized comparator introduces potential for confounding, though propensity score matching will mitigate this. Second, the small sample size limits the precision of cost-effectiveness estimates; results should be interpreted as preliminary evidence to inform future definitive economic evaluations. Third, the 24-week time horizon may not capture the full economic consequences of the intervention if benefits or costs accrue over longer periods. Fourth, the healthcare system perspective excludes productivity losses and informal caregiving costs that may be relevant from a societal perspective. These third and fourth concerns are partly addressed by the secondary long-term Markov extrapolation and the additional societal-perspective analysis described above, which incorporate longer time horizons, productivity losses, and informal caregiver costs; the extrapolated estimates nonetheless remain dependent on modeling assumptions and should themselves be regarded as exploratory. Despite these limitations, this economic evaluation will generate important preliminary evidence to support decision-making regarding BCI technology adoption in stroke rehabilitation.

### 3.11. Implementation Science Framework

Implementation outcomes will be evaluated using the RE-AIM framework (Reach, Effectiveness, Adoption, Implementation, Maintenance), a well-established model for assessing the translation of research interventions into clinical practice [49,50]. This framework enables comprehensive evaluation of factors critical to sustainable implementation of BCI rehabilitation in routine clinical settings (Table 3).

### 3.12. Data Analysis

Data analysis will be performed using Python (version 3.11; Python Software Foundation, Wilmington, DE, USA) programming language with established statistical packages. Descriptive statistics will be expressed as mean and standard deviation (SD) for normally distributed continuous variables or median with interquartile range (IQR) for non-normally distributed data. Given the small sample size characteristic of feasibility studies, non-parametric tests, including the Wilcoxon signed-rank test, will be employed for within-subject comparisons across time points. *p*-values will be corrected for multiple comparisons using the Benjamini–Hochberg False Discovery Rate (FDR) correction, with statistical significance set at alpha = 0.05 [51].

Pre-specified stratified and subgroup analyses will be undertaken. Given potentially divergent recovery trajectories between ischemic and hemorrhagic stroke, feasibility metrics and clinical outcomes will be reported separately for the ischemic and hemorrhagic subgroups, and where cell sizes permit, differences will be explored using exploratory between-subgroup comparisons (Mann–Whitney U tests for continuous outcomes and Fisher’s exact tests for proportions). Outcomes will likewise be summarized by baseline FMA-UE severity stratum (severe, 0–19; moderate-to-mild, 20–47) and, descriptively, by lesion location (cortical, subcortical, brainstem, or cerebellar). Because the feasibility sample (*n* = 12) is not powered for subgroup inference, all stratified analyses are explicitly exploratory and hypothesis-generating; they are intended to estimate the direction and variability of effects by stroke etiology and severity, will be reported with effect estimates and confidence intervals rather than an emphasis on *p*-values, and will inform pre-registered subgroup hypotheses for the future definitive trial.

Based on preliminary data from our meta-analysis of BCI rehabilitation in chronic stroke [24], we expect to observe improvements in FMA-UE scores with a mean change of approximately 5.23 points (standard error 0.70) by the end of 15 training sessions. Historical data from TTSH indicate a mean FMA-UE improvement from 29.1 to 37.4 points (+8.3 points) with standard rehabilitation [40]; thus, BCI may yield an expected total improvement of approximately 13–14 points if effects are additive.

**Interim Analysis:** An interim review will be conducted after enrollment of the first five patients. The interim analysis will evaluate eligibility verification (100% target), informed consent documentation (100% target), serious adverse event rate (0% target), adverse event rate related to training (≤17% target), and dropout rate (less than 20% during training phase). Continuation, modification, or termination of the study will be determined based on these interim metrics.

### 3.13. Safety Monitoring

The Principal Investigator will be responsible for ensuring participant safety on a daily basis. Adverse events will be monitored during every session and throughout the study period of 24 weeks. Serious adverse events (SAEs) include death, life-threatening events, events requiring hospitalization or prolonging existing hospitalization, events resulting in persistent or significant disability, events requiring intervention to prevent permanent impairment, and other medically important events. All SAEs will be reported to the Institutional Review Board per reporting requirements.

**Potential Risks:** Physical risks include skin reactions or transient discomfort to FES pads or EEG headcaps and EEG gel and the theoretical possibility of triggering seizures or computer-related eye fatigue. Additional physical events relevant to acute stroke rehabilitation that will be actively monitored include aggravation of upper limb spasticity, hemiplegic shoulder pain, autonomic symptoms, and falls. Psychological risks include patient fatigue, anxiety or emotional distress, and sleep disturbance, which will be mitigated by allowing breaks between trial blocks and maintaining flexibility in session scheduling.

In recognition that the events listed above are not exhaustive of the adverse reactions relevant to acute stroke rehabilitation, systematic ascertainment of the following will be added to a standardized adverse-event case report form completed at every session and at each follow-up: aggravation of upper limb spasticity (change in Modified Ashworth Scale from baseline), hemiplegic shoulder pain (numeric or visual analogue scale with clinical examination), anxiety or emotional distress (a brief distress screen, with escalation to formal assessment as indicated), sleep disturbance, autonomic symptoms, falls, and fatigue. Each event will be recorded with its onset, severity, duration, relatedness to the intervention (graded unrelated, unlikely, possible, probable, or definite), action taken, and outcome. Serious adverse events will be defined per International Council for Harmonisation Good Clinical Practice criteria (death; life-threatening events; hospitalization or its prolongation; persistent or significant disability or incapacity; or other medically important events) and reported to the SingHealth Centralised Institutional Review Board within the mandated timelines using the board’s serious-adverse-event reporting forms, with parallel notification of the Principal Investigator and the study sponsor; an independent clinician not involved in intervention delivery will adjudicate the relatedness of all serious events. Recognizing that many clinically important events in this population are systemic rather than device-related, non-BCI-specific adverse events—recurrent stroke, seizures, sepsis, cardiac events, venous thromboembolism, autonomic dysfunction, and neuropsychiatric symptoms such as depression and anxiety—will additionally be ascertained by review of each participant’s electronic discharge summary rather than via a separate dedicated checklist given resource constraints.

### 3.14. Ethical Considerations

This study will be conducted in accordance with the ethical principles of the Declaration of Helsinki and consistent with Good Clinical Practice (GCP) guidelines. The protocol has been approved by the SingHealth Centralised Institutional Review Board (CIRB) (#2024-3186) and mutually recognized by the National Health Group Domain Specific Review Board (DSRB). The trial will be registered on ClinicalTrials.gov prior to participant enrollment. Written informed consent will be obtained from all participants or their legal representatives prior to enrollment. Given that the study requires preserved cognition for active BCI participation, patients unable to provide informed consent due to neurological injury will be excluded. All study-related documents, data, and information will be kept strictly confidential. Data used in analyses will be anonymized without reference to specific participant names. All records will be retained for at least 15 years after study termination.

## 4. Discussion

This manuscript presents a prospective feasibility study protocol examining the implementation of BCI-based upper limb neurorehabilitation during the early subacute phase following stroke, with an integrated implementation science and economic evaluation [31,32]. The novelty of this study lies in addressing a key evidence gap by evaluating closed-loop BCI rehabilitation during the acute and subacute post-stroke period (2–12 weeks), when neuroplasticity is theoretically the greatest, whereas most prior BCI studies have focused on chronic stroke patients who have already plateaued after conventional rehabilitation [24]. Beyond assessing efficacy, it advances the field toward real-world clinical adoption by evaluating implementation within the acute inpatient rehabilitation setting, incorporating a preliminary cost analysis, and investigating EEG-based biomarkers of recovery to inform future adaptive, biomarker-guided BCI therapies.

We highlight the following limitations: Without a concurrent control group, any observed changes cannot be definitively attributed to the BCI intervention versus spontaneous recovery in the acute/subacute phase. This is a particularly important consideration in the 2–12-week window, when substantial spontaneous neurological recovery occurs and follows broadly predictable, proportional-recovery-type trajectories, making the separation of intervention-related gain from natural recovery intrinsically difficult in a single-arm design. To characterize and bound this confounder, we will (i) benchmark the observed FMA-UE and ARAT trajectories against the spontaneous-recovery estimates derived from the propensity-matched historical controls and from published early-recovery models, (ii) examine whether within-training electrophysiological change (ERD/ERS and decoding accuracy) tracks clinical change more closely than the passage of time alone would predict, and (iii) present sensitivity analyses that attribute conservative proportions of the observed gain to spontaneous recovery. These steps cannot substitute for a concurrent randomized control arm, which remains the definitive solution and is planned for the subsequent trial. The use of historical controls for economic evaluation introduces potential residual confounding. However, this study is intended as a pragmatic feasibility study that would in future support a larger study. The small sample size (*n* = 12) is appropriate for feasibility but limits precision, subgroup analysis, and generalizability, meaning that all findings are intended as hypothesis-generating to inform a future adequately powered trial. A specific consideration in this early phase is patient selection: motor imagery is attention-dependent, and the fatigue, reduced concentration, and medical instability that are common soon after stroke may limit who can engage with the paradigm. Determining which acute and early subacute patients can tolerate and reliably operate the BCI is therefore an explicit feasibility output of this study and will be used to refine eligibility criteria for a future definitive trial.

For transparency, we set out each principal limitation together with the specific design improvement it motivates for a future adequately powered study: (1) the single-arm design will be replaced by a randomized, concurrently controlled trial; (2) reliance on historical controls, with its residual and temporal confounding, will be addressed through prospective concurrent recruitment and, for the economic analysis, contemporaneous comparators; (3) the small feasibility sample will be superseded by a sample size formally powered on the FMA-UE minimal clinically important difference and the variance estimates generated here; (4) the unequal rehabilitation dose will be resolved by a time-equated active or attention-controlled comparator; (5) the 24-week within-trial horizon will be extended by the long-term Markov extrapolation and by the additional 12-month clinical follow-up; (6) the incomplete separation of stroke etiologies will be addressed by pre-registering etiology- and severity-stratified subgroup hypotheses; and (7) the exploratory electrophysiological biomarker and action-observation predictors identified here will be advanced as pre-specified candidate predictors of response. Collectively, these refinements define a clear translational pathway from the present feasibility study to a definitive effectiveness and cost-effectiveness trial.

The findings from this study will have several important implications. Clinically, demonstration of feasibility and safety will support the integration of BCI technology into early stroke rehabilitation pathways. The identification of MI-EEG correlates with clinical efficacy measures will inform early treatment-related plasticity effects. A comprehensive economic evaluation will inform preliminary justification of the additional device and set-up costs and efforts required. The preliminary economic data will help healthcare administrators and policymakers understand the potential value proposition of BCI technology in stroke rehabilitation to inform resource allocation decisions.

Finally, from a health-service and economic standpoint, a plausible future application lies in telemedicine and home- or community-based delivery. The scenario analysis modeling partial substitution and reduced supervision described above is a first step in this direction: remote or minimally supervised BCI rehabilitation, with tele-supervised set-up and remote monitoring of adherence, decoding performance, and safety, could substantially improve access and reduce per-session therapist and facility costs while extending assessment and follow-up beyond the inpatient setting. Establishing the feasibility, safety, and reliability of supervised inpatient delivery in the present study is a necessary precursor to evaluating such tele-rehabilitation models, which we identify as a priority for subsequent research.

## Figures and Tables

**Table 1:** Outcome measures and assessment instruments.

Outcome Measure	Score Range	Description
Motor Impairment and Upper Limb Function		
Fugl–Meyer Assessment—Upper Extremity (FMA-UE)	0–66	Standardized, highly inter-reliable tool for the assessment of motor impairment in stroke-related hemiplegia; higher scores indicate less impairment [34]
Action Research Arm Test (ARAT)	0–57	Assessment of upper limb function including grasp, grip, pinch, and gross motor movements
**Functional, Quality-of-Life, Usability, Safety, and Economic Measures**		
Modified Barthel Index (MBI)	0–100	Activities of daily living independence measure
Functional Independence Measure (FIM)	18–126	Activities of daily living independence measure, level of care/dependency
EQ-5D-5L; Stroke Impact Scale (SIS 3.0) domains 1, 5, 7, 8, 9. e.g.; domains 1 (arm), 5 (ADL), 7 (hand), 8 (societal activities), 9 (Stroke recovery rating)	Index 0–1; VAS 0–100	Health-related quality of life assessment for QALY calculation Minimum to maximum scores/domain: 4–10 (domain 1), 10–50 (domain 5), 5–25 (domain 7), 8–40 (domain 8), 0–100 (domain 9). Higher scores indicating better function
System Usability Scale (SUS)	0–100	Subjective assessment of BCI system usability (self-rated) > 70/100 indicates good usability
Pain-Visual Analogue Scale (VAS)	0–10	Subjective assessment of pain
Modified Ashworth Scale (MAS)	0, 1, 1+, 2,3,4	Clinical assessment of spasticity in elbow and finger flexors (scale is non-ordinal)
Health economic evaluation	Not applicable	Incremental cost-utility analysis: incremental cost-effectiveness ratio (cost per quality-adjusted life year) and cost per one-point improvement in FMA-UE

**Table 2:** Components of economic evaluation.

Cost Category	Components
BCI Intervention Costs (Intervention Group Only)	
Equipment costs	recoveriX system (purchase/rental/depreciation), EEG cap, FES unit
Consumables	EEG gel, FES electrode pads, disinfection disposables (alcohol swabs, gloves, hand rubs) per session
Incremental therapist time	Additional 30 min OT time per session (BCI group receives 90 min total vs. standard 60 min OT)
Training costs	Staff training and certification (allocated per patient)
**Healthcare Utilization Costs (Both Groups)**	
Inpatient rehabilitation	Length of stay, ward type (Class B2/C subsidized wards as reference case), average daily bed cost, total OT/PT sessions
Outpatient services	Rehabilitation clinic visits, community therapy sessions (post-discharge)
Readmissions	Any stroke-related or rehabilitation-related hospital readmissions within 24 weeks

**Table 3:** RE-AIM framework assessment domains and variables.

Domain	Variables to Be Collected
Reach	Percentage excluded by eligibility criteria; proportion of eligible patients who participate; characteristics of participants versus non-participants
Effectiveness	Primary outcome measures (FMA-UE, ARAT); quality of life outcomes; adverse events; subgroup analyses; cost-effectiveness metrics
Adoption	Setting exclusion rates; patient disposition; staff participation rates; characteristics of participating versus non-participating staff
Implementation	Protocol adherence and fidelity rates; adaptations made during study; time and resource costs; consistency across staff and settings
Maintenance	Long-term primary outcomes (6+ months); sustained subgroup effects; organizational adoption sustainability

## Data Availability

The datasets generated during the current study will be available from the corresponding author on reasonable request.
